# Mitochondrial Dysfunction Mediated by Poly(ADP-Ribose) Polymerase-1 Activation Contributes to Hippocampal Neuronal Damage Following Status Epilepticus

**DOI:** 10.3390/ijms18071502

**Published:** 2017-07-12

**Authors:** Yi-Chen Lai, J. Scott Baker, Taraka Donti, Brett H. Graham, William J. Craigen, Anne E. Anderson

**Affiliations:** 1 Departments of Pediatrics, Baylor College of Medicine, Houston, TX 77030, USA; ylai@bcm.edu (Y.-C.L.); baylorscott2001@yahoo.com (J.S.B.); 2 Departments of Molecular and Human Genetics, Baylor College of Medicine, Houston, TX 77030, USA; tdonti@ggc.org (T.D.); bregraha@iu.edu (B.H.G.); wcraigen@bcm.edu (W.J.C.); 3 Departments of Neurology, Baylor College of Medicine, Houston, TX 77030, USA; 4 Departments of Neuroscience, Baylor College of Medicine, Houston, TX 77030, USA

**Keywords:** hippocampus, mitochondria, neuronal damage, poly(ADP-ribose) polymerase-1, status epilepticus

## Abstract

Mitochondrial dysfunction plays a central role in the neuropathology associated with status epilepticus (SE) and is implicated in the development of epilepsy. While excitotoxic mechanisms are well-known mediators affecting mitochondrial health following SE, whether hyperactivation of poly(ADP-ribose) polymerase-1 (PARP-1) also contributes to SE-induced mitochondrial dysfunction remains to be examined. Here we first evaluated the temporal evolution of poly-ADP-ribosylated protein levels in hippocampus following kainic acid-induced SE as a marker for PARP-1 activity, and found that PARP-1 was hyperactive at 24 h following SE. We evaluated oxidative metabolism and found decreased NAD^+^ levels by enzymatic cycling, and impaired NAD^+^-dependent mitochondrial respiration as measured by polarography at 24 h following SE. Stereological estimation showed significant cell loss in the hippocampal CA_1_ and CA_3_ subregions 72 h following SE. PARP-1 inhibition using *N*-(6-Oxo-5,6-dihydro-phenanthridin-2-yl)- *N*,*N*-dimethylacetamide (PJ-34) in vivo administration was associated with preserved NAD^+^ levels and NAD^+^-dependent mitochondrial respiration, and improved CA_1_ neuronal survival. These findings suggest that PARP-1 hyperactivation contributes to SE-associated mitochondrial dysfunction and CA_1_ hippocampal damage. The deleterious effects of PARP-1 hyperactivation on mitochondrial respiration are in part mediated through intracellular NAD^+^ depletion. Therefore, modulating PARP-1 activity may represent a potential therapeutic target to preserve intracellular energetics and mitochondrial function following SE.

## 1. Introduction

Status epilepticus (SE), defined as seizure activities lasting greater than 30 min [1], is associated with hippocampal neuronal damage and implicated in the development of hippocampal sclerosis and epilepsy [2,3,4]. Several mechanisms contribute to SE-induced neuronal damage and cell death with mitochondrial dysfunction representing an important and well-known pathological feature [3,5,6,7,8]. Furthermore, impaired mitochondrial function has emerged as a common pathological finding in experimental and human epilepsy [9,10,11] suggesting that persistent mitochondrial dysfunction may play a role in the neuropathology of recurrent, unprovoked seizures. SE can lead to activation of *N*-methyl-d-aspartate (NMDA) receptors, which in turn increases intra-mitochondrial Ca^2+^ accumulation, as well as oxidative and nitrosative stress. These pathological processes ultimately contribute to impaired oxidative phosphorylation and loss of mitochondrial membrane potential and structural integrity [3,7,8,9,10,12]. Furthermore, NMDA-mediated nitric oxide synthase activation and subsequent oxidative stress have been shown to induce poly(ADP-ribose) polymerase-1 (PARP-1) activity [13,14,15], which also contributes to mitochondrial failure associated with NMDA-mediated excitotoxicity [14,16].

Indeed, increased PARP-1 activity has been observed following continuous epileptiform discharges in vitro, and in focal excitotoxic injury in vivo [17,18,19,20]. Pharmacological PARP-1 inhibition in these studies decreases brain lesion volume and neuronal loss [18,19,20] suggesting a deleterious role of PARP-1 in SE-associated neuronal injury. The poly-ADP-ribosylation reaction mediated by PARP-1 is an energy-requiring post-translational modification, converting a massive quantity of nicotinamide adenine dinucleotide (NAD^+^) to linear and branched poly-ADP-ribose polymers (pADPr) [21]. Therefore, PARP-1 activation classically has been associated with intracellular NAD^+^ depletion and mitochondrial dysfunction leading to cell death [14,22,23]. Additionally, PARP-1 can adversely affect mitochondrial function through free pADPr molecules as a direct inducer of mitochondrial failure [24,25], inhibition of glycolysis [22,26,27,28], and possibly alteration of mitochondrial protein function by post-translational modification [29]. Although the detrimental effects of PARP-1 activation on mitochondrial function have been well described in neurodegenerative disease, cerebral ischemia, and trauma [23,29,30,31], whether PARP-1 activation contributes to the mitochondrial dysfunction commonly associated with SE, and the mechanisms by which PARP-1 mediates mitochondrial failure in SE are unknown.

In this study we investigated whether PARP-1 activation was associated with the canonical intracellular NAD^+^ depletion-mitochondrial failure pathway and contributed to hippocampal neuronal damage in a model of chemoconvulsant-induced SE using kainic acid [32]. We observed PARP-1 activation in the hippocampus at 24 h following SE. PARP-1 activation was associated with decreased intracellular NAD^+^ levels, impaired NAD^+^-dependent mitochondrial respiration and hippocampal CA_1_ damage. PARP-1 did not affect the function of individual electron transport chain complexes, suggesting that the impairment in NAD^+^-dependent mitochondrial respiration likely reflects decreased availability of NAD^+^ as a cofactor.

## 2. Results

### 2.1. Increased PARP-1 Activity in the Hippocampus Following SE

**Figure 1:**
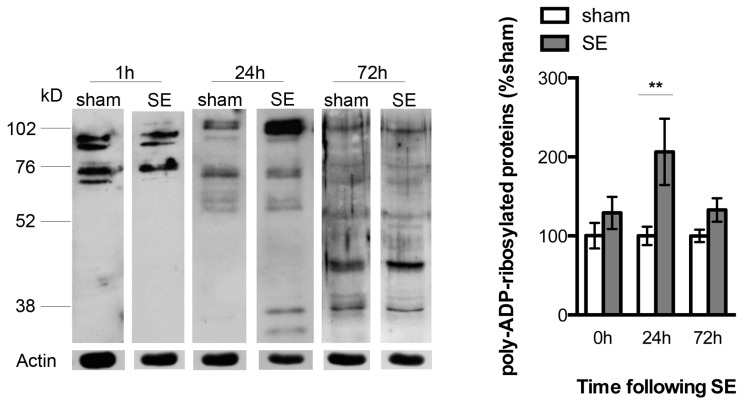
Representative western blots and the quantification of poly-ADP-ribosylated protein bands from the hippocampal homogenates of the sham and SE (status epilepticus) animals. The SE animals exhibited increased immunoreactivity and poly-ADP-ribosylated protein bands at 24 h following SE, suggesting an increase in PARP-1 activity (*n* = 6–8/group, ** *p* < 0.01).

We evaluated PARP-1 activity in the hippocampus following SE by assessing the amount of poly-ADP-ribosylated proteins in the hippocampal whole cell homogenates using western blotting. Compared with sham animals, the SE animals exhibited increased immunoreactivity for the poly-ADP-ribosylated proteins in hippocampal homogenates at 24 h following SE (100 ± 11.68% vs. 206.4 ± 41.89%, sham vs. SE, *n* = 6–8/group, *p* < 0.01, Figure 1). By 72 h following SE, increases in poly-ADP-ribosylated protein immunoreactivity was no longer evident and the levels were comparable between the sham and the SE animals (100 ± 7.98% vs. 109.1 ± 11.42%, sham vs. SE, *n* = 6/group, Figure 1).

### 2.2. PARP-1 Activation Following SE Was Associated with Intracellular NAD^+^ Depletion

**Figure 2:**
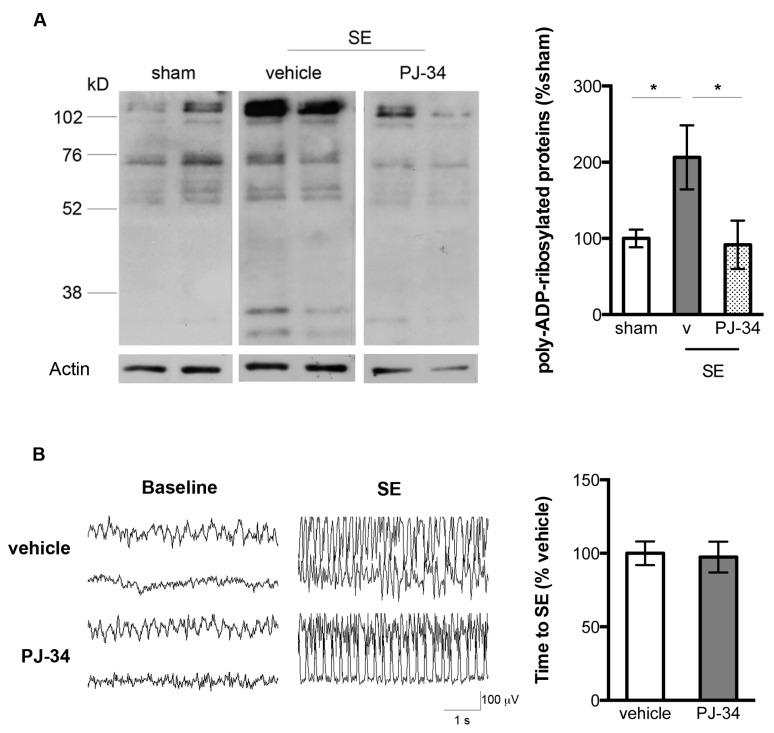
(**A**) Representative western blots (2 animals per treatment group) and quantification of poly-ADP-ribosylated proteins from the hippocampal homogenates of the sham, vehicle-treated SE, and PJ-34 treated SE animals at 24 h following SE. The vehicle-treated SE animals exhibited increased immunoreactivity of the poly-ADP-ribosylated proteins. The increased immunoreactivity was attenuated in the PJ-34 treated SE animals (*n* = 6–7/group, * *p* < 0.05); (**B**) Vehicle and PJ-34 treated animals exhibited similar EEG patterns at baseline and during SE (*n* = 5/group) and comparable latency to SE (*n* = 12–13/group).

In order to evaluate the role of PARP-1 activation in SE-associated mitochondrial dysfunction and hippocampal damage, we modulated PARP-1 activity using an inhibitor *N*-(6-Oxo-5,6-dihydro-phenanthridin-2-yl)-*N*,*N*-dimethylacetamide (PJ-34). Again, western blotting of the poly-ADP-ribosylated proteins demonstrated significantly increased immunoreactivity in the vehicle-treated SE animals at 24 h following SE as compared with the sham animals (100 ± 11.68% vs. 206.4 ± 41.89%, sham + vehicle vs. SE + vehicle, *n* = 6–8/group, *p* < 0.05, Figure 2A). PJ-34 administration prior to the induction of SE significantly attenuated the levels of immunoreactive protein bands following SE indicating fewer poly-ADP-ribosylated proteins (206.4 ± 41.89% vs. 91.56 ± 31.77%, SE + vehicle vs. SE + PJ 34, *n* = 6–7/group, *p* < 0.05, Figure 2A). Inhibition of PARP-1 had no appreciable effects on brain activities based on the baseline electroencelographic (EEG) patterns (Figure 2B). In addition, there was no effect on the latency to SE when comparing between vehicle and PJ-34 treated animals (57.57 ± 4.51 min vs. 49.09 ± 4.49 min, SE + vehicle vs. SE + PJ-34, *n* = 12–13/group, Figure 2B) suggesting that PARP-1 inhibition did not alter animals’ susceptibility to kainic acid-induced SE.

**Figure 3:**
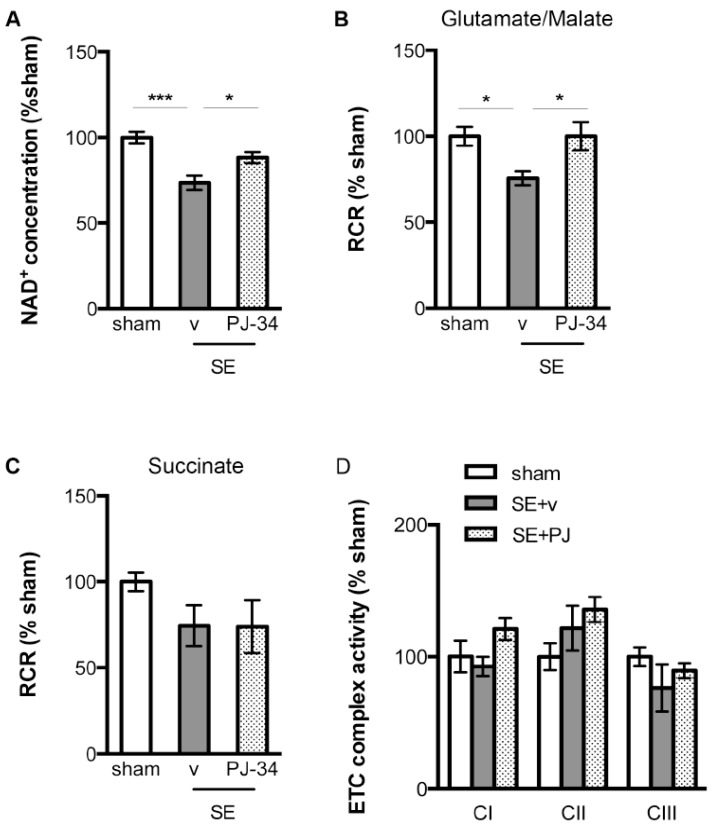
(**A**) Twenty-four hours following SE, hippocampal whole cell homogenate of the vehicle-treated SE animals contained less NAD^+^ as compared with the sham animals. PJ-34 ameliorated intracellular NAD^+^ depletion associated with SE (*n* = 9/group, * *p* < 0.05, *** *p* < 0.001); (**B**) Twenty-four hours following SE, mitochondria of the vehicle-treated SE animals exhibited decreased O_2_ consumption (respiratory control ratio (RCR)) as compared with the sham animals when mitochondria were provided with Complex I substrates (glutamate/malate). PJ-34 preserved mitochondrial O_2_ consumption using Complex I substrates (*n* = 6/group, * *p* < 0.05); (**C**) No differences in O_2_ consumption were observed between the sham animals and either vehicle-treated or PJ-34 treated SE animals (*n* = 6/group) when mitochondria were provided with Complex II substrate (succinate); (**D**) The intrinsic activities of Complex I, II, and III were comparable between the sham animals and either vehicle-treated or PJ-34 treated animals 24 h following SE (*n* = 4/group). RCR: respiratory control ratio. ETC: electron transport chain. CI: Complex I. CII: Complex II. CIII: Complex III.

Because poly-ADP-ribosylation reaction mediated by PARP-1 converts a massive quantity of NAD^+^ to linear and branched poly-ADP-ribose polymers (pADPr) [21], we investigated whether increased PARP-1 activity in the hippocampus following SE was associated with decreased intracellular NAD^+^ content; and whether PARP-1 inhibition by PJ-34 would preserve NAD^+^ levels. Concurrent with the increased PARP-1 activity, there were significantly decreased levels of total NAD^+^ in the hippocampal whole cell homogenate from vehicle-treated SE animals at 24 h following SE as compared with the sham cohort (100 ± 3.35% vs. 73.62 ± 4.13%, sham + vehicle vs. SE + vehicle, *n* = 9/group, *p* < 0.001, Figure 3A). PARP-1 inhibition by PJ-34 was associated with the preservation of intracellular NAD^+^ reflected in higher intracellular NAD^+^ levels in the PJ-34 treated SE animals as compared with the vehicle-treated SE animals (73.62 ± 4.13% vs. 88.26 ± 3.17%, SE + vehicle vs. SE + PJ-34, *n* = 9/group, *p* < 0.05, Figure 3A).

### 2.3. PARP-1 Activation Following SE Was Associated Impaired NAD^+^-Dependent Mitochondrial Respiration

We evaluated whether the observed PARP-1 activation and intracellular NAD^+^ depletion following SE were associated with mitochondrial dysfunction. The integrity of mitochondrial respiration was assessed using respiratory control ratio (RCR), a ratio of O_2_ consumption rates by the mitochondria in the presence and absence of substrates that are required for oxidative phosphorylation [33]. Oxidative phosphorylation can be initiated by providing substrates for Complex I of the electron transport chain (malate/glutamate), which requires NAD^+^ as a cofactor. Vehicle-treated SE animals exhibited significantly decreased RCR as compared with the sham animals in the presence of Complex I substrates (100 ± 5.44% vs. 75.60 ± 4.00%, sham + vehicle vs. SE + vehicle, *n* = 6/group, *p* < 0.05, Figure 3B). PARP-1 inhibition with PJ-34 resulted in preserved RCR following SE as compared with the vehicle-treated SE animals (75.60 ± 4.00% vs. 100.1 ± 8.19%, SE + vehicle vs. SE + PJ-34, *n* = 6/group, *p* < 0.05, Figure 3B).

Oxidative phosphorylation can also be initiated by providing substrates for Complex II of the electron transport chain (succinate), which requires FAD^2+^ as cofactor. There were no statistically significant differences in the RCR values from sham, vehicle-treated SE, and PJ-34 treated SE animals when mitochondria were supplied with Complex II substrates (100.0 ± 5.47%, 74.37 ± 11.98%, 73.82 ± 15.38%, sham + vehicle, SE + vehicle, SE + PJ-34, *n* = 6/group, Figure 3C).

We then investigated whether PARP-1 activation could directly affect the function of individual electron transport chain complexes, particularly Complex I. When assessing individual electron transport chain complexes by spectrophotometric kinetic assays, we found that in the presence of the exogenous NAD^+^, the intrinsic Complex I activity of the vehicle-treated SE animals was similar to the sham and PJ-34 treated SE animals (Figure 3D). Furthermore, no differences in the intrinsic Complex II and Complex III activities were observed between the sham, vehicle-treated SE, and PJ-34 treated SE animals (Figure 3D). Together, these finding suggest that PARP-1 activation following SE did not alter the function of individual transport chain complexes. Therefore, the observed impairment in NAD^+^-dependent mitochondrial respiration likely reflects decreased availability of NAD^+^ as a cofactor.

### 2.4. PARP-1 Activation Contributed to SE-Associated Hippocampal CA_1_ Neuronal Damage

**Figure 4:**
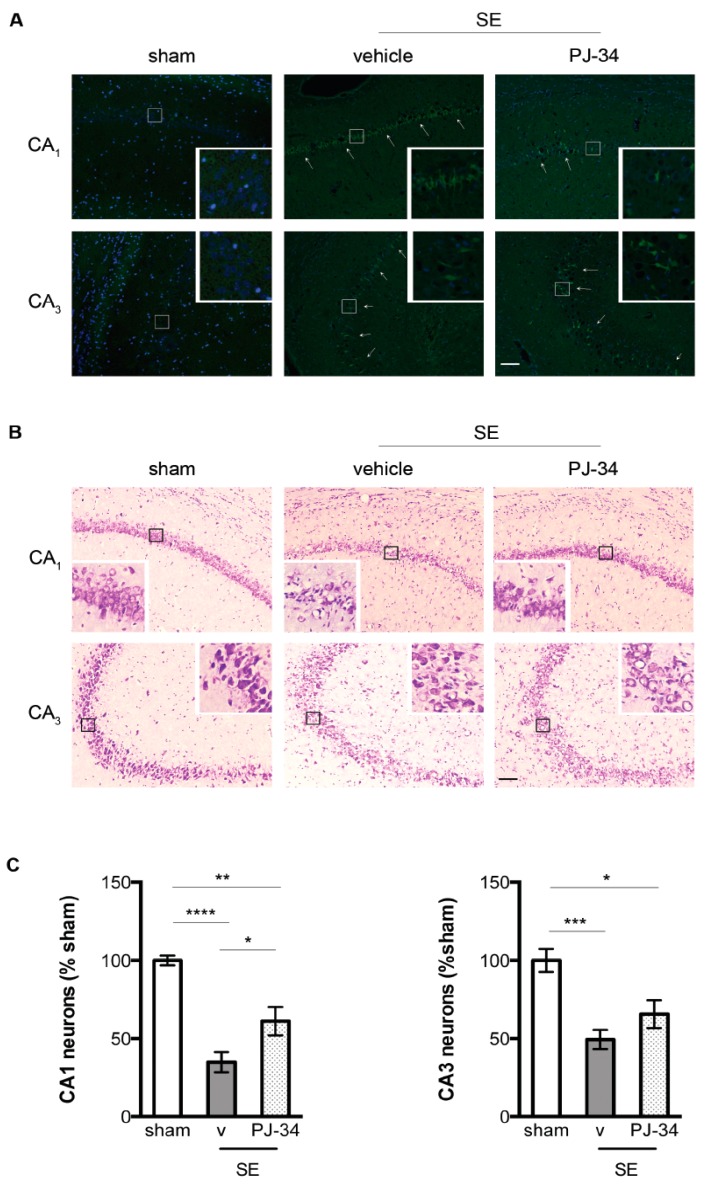
(**A**) Representative Fluoro-Jade B (FJ-B) staining of the sham, vehicle-treated SE and PJ-34 treated SE animals at 72 h following SE. Vehicle and PJ-34 treated animals exhibited increased FJ-B staining (white arrows) in the CA_1_ and CA_3_ regions of the hippocampus following SE as compared with the sham animals. PJ-34 treated SE animals exhibited decreased FJ-B staining in the CA1_1_ region as compared with the vehicle-treated SE animals (*n* = 6/group); (**B**) Representative Nissl staining of the sham, vehicle-treated SE, and PJ-34 treated SE animals demonstrating thinning and disorganization in the stratum pyramidale of the CA_1_ and CA_3_ regions of the vehicle treated animals 72 h following SE (*n* = 6/group). PJ-34 preserved stratum pyramidale of the CA_1_ region while having no effects on the CA_3_ region; (**C**) Stereological estimation revealed significantly fewer CA_1_ neurons in the vehicle-treated SE animals 72 h following SE. PARP-1 inhibition by PJ-34 was associated with more CA_1_ neurons as compared with the vehicle-treated animals (*n* = 6/group, * *p* < 0.05, ** *p* < 0.01, **** *p* < 0.0001). In contrast, SE was associated with significantly fewer CA_3_ neurons in both vehicle-treated and PJ-34 treated animals 72 h following SE (*n* = 6/group, * *p* < 0.05, *** *p* < 0.001). Insert images represent higher magnification of the CA_1_ and CA_3_ pyramidal neurons from the areas highlighted in (**A**,**B**) (squares). Scale bar = 100 microns.

Because mitochondrial dysfunction contributes significantly to SE-associated neuronal damage [3,5,6,7] and modulating PARP-1 activity preserved mitochondrial respiration following SE in our study, we examined whether this preservation of mitochondrial respiration was associated with improved hippocampal neuronal survival. At 72 h following SE, Fluoro-Jade B (FJ-B) staining revealed increased FJ-B positive cells in the stratum pyramidale of the CA_1_ and CA_3_ hippocampal subfields in the vehicle-treated SE animals as compared with the sham animals suggesting increased neuronal damage (Figure 4A). Accordingly, H&E staining revealed disorganized stratum pyramidale with variable stages of cell death represented by degenerating neurons, pyknotic or swollen cells, and vacuoles in the vehicle-treated SE animals (Figure 4B).

Stereological estimates of the hippocampal neurons revealed significant cell loss in the vehicle-treated SE animals 72 h following SE (100 ± 3.03% vs. 34.92 ± 6.46% sham + vehicle vs. SE + vehicle, *n* = 6/group, *p* < 0.0001, Figure 4C). PARP-1 inhibition decreased SE-induced damage in the CA_1_ hippocampal subfield as reflected in fewer FJ-B positive cells (Figure 4A) and damaged neurons in the CA_1_ region (Figure 4B) of the PJ-34 treated SE animals, as well as more CA_1_ neurons in the PJ-34 treated SE animals by stereological estimation (34.92 ± 6.46% vs. 61.18 ± 9.11%, SE + vehicle vs. SE + PJ-34, *n* = 6/group, *p* < 0.05, Figure 4C). PARP-1 inhibition did not improve neuronal survival in the CA_3_ region, reflected in comparable FJ-B and H&E staining (Figure 4A,B), and stereological estimation between the vehicle and PJ-34 treated SE animals (100.0 ± 7.15% vs. 49.44 ± 6.17% vs. 65.69 ± 8.83%, sham + vehicle, SE + vehicle, SE + PJ-34, *n* = 6/group, Figure 4C).

## 3. Discussions

Mitochondrial dysfunction represents an important neuropathology of cell damage and death following SE [3,5,6,7,8]. While NMDA-mediated excitotoxic mechanisms involving dysregulated Ca^2+^ homeostasis, oxidative, and nitrosative stress are well-known contributors of seizure-induced mitochondrial injury [3,7,8,9,10,12], the role of PARP-1 activation on mitochondrial dysfunction in SE is less understood. Increased PARP-1 activity and pADPr levels have been observed under excitotoxic conditions and contribute to mitochondrial failure [15,16,22,25,26]. Furthermore, PARP-1 activation has been associated with decreased NAD^+^ and ATP levels following kainic acid-induced damage to the striatum [18], supporting a deleterious effect of PARP-1 on cellular energetics associated with excitotoxic injury. Here we built upon these observations and demonstrated that PARP-1 was active following kainic acid-induced SE as reflected in the increased immunoreactivity of the poly-ADP-ribosylated proteins. Inhibiting PARP-1 activation preserved NAD^+^ levels and mitochondrial respiration in the SE animals. Together, our findings suggest that PARP-1 activation may contribute to the mitochondrial dysfunction and impaired cellular energetics that occur following SE.

Our findings also provide a candidate mechanism by which PARP-1 activation leads to mitochondrial dysfunction. We found that inhibiting PARP-1 activity preserved intracellular NAD^+^ levels and NAD^+^-dependent mitochondrial respiration, while having no effects on the FAD^2+^-dependent mitochondrial respiration or the function of individual electron transport chain complexes. These findings suggest that PARP-1 activation following SE may lead to decreased NAD^+^ availability, which in turn contributes to mitochondrial dysfunction. Decreased intracellular NAD^+^ levels potentially can affect mitochondrial function through multiple mechanisms. NAD^+^ serves as a cofactor for several glycolytic enzymes. Accordingly, PARP-1 mediated NAD^+^ depletion has been shown to inhibit glycolysis in vitro, thereby decreasing available substrates for oxidative phosphorylation in the mitochondria [22,26]. Alternatively, Complex I of the electron transport chain also requires NAD^+^ as a cofactor. Therefore, NAD^+^ depletion due to PARP-1 activation could directly impact mitochondrial respiration when Complex I substrates are utilized. In this study, we measured total cellular NAD^+^ levels, therefore we were unable to assess whether decreased NAD^+^ levels affected primarily glycolysis, mitochondrial respiration, or both. Future studies are needed to further delineate the relative impact of PARP-1 activation and NAD^+^ depletion in different cellular compartments.

In this study, we have demonstrated that the canonical PARP-1 activation-intracellular energy depletion-mitochondrial dysfunction pathway is active and contributes to hippocampal neuronal damage following SE. However, impaired cellular energetics represents only one of many mechanisms through which PARP-1 can adversely affect mitochondrial health. For instance, free pADPr molecules generated from PARP-1 activation are biologically active through noncovalent interactions with target proteins [34]. Noncovalent binding of pADPr to hexokinase, a rate-limiting glycolytic enzyme, can inhibit glycolysis [27], which may result in subsequent mitochondrial dysfunction. Furthermore, free pADPr molecules have been implicated in directly inducing mitochondrial failure and releasing apoptosis-inducing factors from the mitochondria, which lead to subsequent neuronal death [24,25]. Whether the free pADPr molecules generated from PARP-1 activation also contribute to mitochondrial dysfunction and cell death following SE remains to be determined.

We report in this study that PARP-1-dependent mitochondrial dysfunction, a well-described pathological mechanism associated with NMDA mediated excitotoxicity, contributes to hippocampal CA_1_ damage following SE. A possible mechanism by which PARP-1 activation leads to mitochondrial dysfunction is through its effects on the intracellular energetics as represented by decreased NAD^+^ levels. Our findings represent only one of multiple mechanisms through which PARP-1 can adversely affect mitochondrial health and neuronal survival. Therefore, additional studies are required to further characterize the effects of PARP-1 activation on mitochondrial dysfunction and neuronal injury following SE.

## 4. Materials and Methods

### 4.1. Kainic Acid-Induced SE

**Figure 5:**
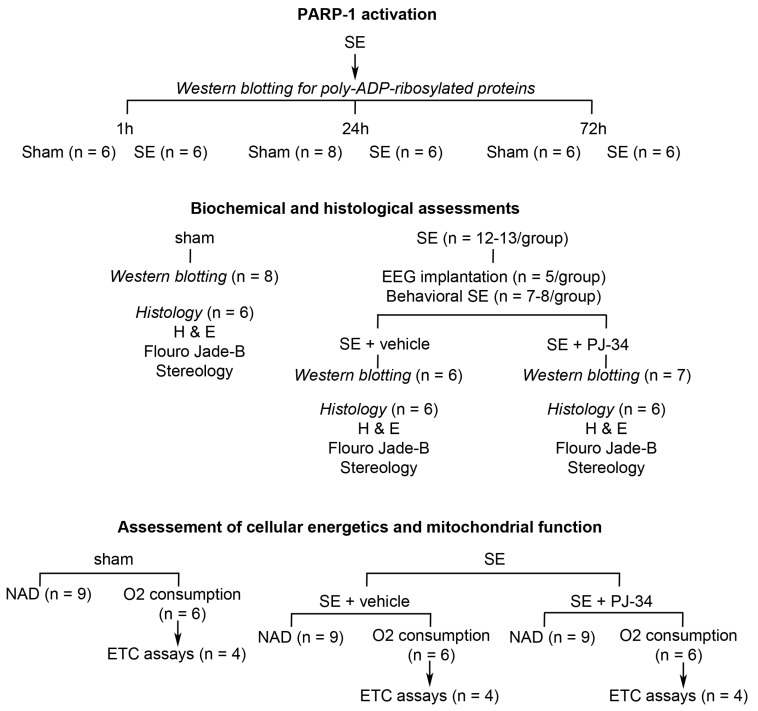
Experimental design.

All animal experiments were approved by the institutional committee on animal care and conformed to the guidelines of the National Institute of Health for the care and use of the laboratory animals (Figure 5). Intraperitoneal kainic acid (15 mg/kg, i.p.) was administered to Sprague–Dawley rats (120–180 g) to induce SE. Behavioral seizures and SE were monitored using the Racine scale [35]. Following kainate administration, animals developed generalized convulsive SE. They remained in SE for one hour, followed by pentobarbital administration (20 mg/kg, i.p.) to terminate seizures. For the pharmacological studies, the animals received a daily dose of either vehicle (normal saline) or PARP-1 inhibitor *N*-(6-Oxo-5,6-dihydro-phenanthridin-2-yl)-*N*,*N*-dimethylacetamide (PJ-34, 15 mg/kg) for two days before, and one dose on the day of the experiment 30 min prior to seizure induction.

### 4.2. Stereotaxic EEG Implantation

In a subset of animals, surface and depth EEG electrodes were implanted at the following coordinates (from Bregma) based on Paxinos & Watson’s brain atlas for rats with modifications: *Hippocampus:* Left: +3.4 mm lateral, −3.6 mm posterior, −3.4 mm depth. Right: −3.4 mm lateral, −3.6 mm posterior, −3.4 mm depth. *Surface:* Left: +3.4 mm lateral, −3.2 mm posterior. Right: −3.4 mm lateral, −3.2 mm posterior. Following one week of recovery, the animals were treated with either PJ-34 or vehicle. Thirty minutes of baseline EEG recordings were obtained immediately prior to the kainic acid administration and throughout the experiment.

### 4.3. pADPr Western Blotting

Animals were sacrificed and hippocampi dissected at 1 h, 24 h and 72 h following SE. A hippocampal homogenate was obtained as previously described [36]. For western blotting, 50 μg of proteins were separated by SDS-PAGE and probed with anti-pADPr antibody (Trevigen, Gaithersburg, MD, USA) at a 1:500 dilution 4 °C overnight. The relative abundance of pADPr-modified proteins in each sample was determined by densitometry (ImageJ, NIH, Bethesda, MD, USA) and expressed as relative optical density (ROD).

### 4.4. Mitochondrial Oxygen Consumption

Hippocampal mitochondria were isolated by differential centrifugation 24 h following SE. Two hippocampi were homogenized in 10 mL of the isolation buffer (210 mM mannitol, 70 mM sucrose, 1 mM EGTA, 5 mM HEPES pH 7.5, 0.5% bovine serum albumin) and centrifuged at 1500× *g* for 5 min at 4 °C. The supernatant was subsequently centrifuged at 8000× *g* for 15 min at 4 °C. The resulting pellet was re-suspended in 25 mL of the isolation buffer and centrifuged at 8000× *g* for 15 min at 4 °C. The pellet was re-suspended in 50 μL of the isolation buffer and the protein concentration determined by the Bradford method. Oxygen consumption was measured using a Clark-type electrode. Ten microliters of mitochondrial proteins were added to the respiration chamber containing the respiration buffer (225 mM mannitol, 75 mM sucrose, 10 mM KCl, 10 mM Tris-HCl, pH 7.2, 5 mM KH_2_PO_4_, pH 7.2), 125 nM ADP, and either Complex I (5 mM glutamate, 5 mM malate) or Complex II (5 mM succinate) substrate. The mitochondria were fully depolarized by 6.5 mM of 2,4-dinitrophenol (DNP) at the end of each oxygen consumption measurement.

### 4.5. Assessment of Electron Transport Chain Complex Activity

The spectrophotometric kinetic assays were performed at 30 °C in a volume of 175 μL using a monochromator microplate reader (Tecan M200). Complex I activity (NADH:ubiquinone oxidoreductase) was determined by measuring the oxidation of NADH at 340 nm using ferricyanide as the electron acceptor in a reaction mixture of 25 mM potassium phosphate (pH 7.5), 0.2 mM NADH, and 1.7 mM potassium ferricyanide. Complex II activity (succinate dehydrogenase) was determined by measuring the reduction of the artificial electron acceptor 2,6-dichlorophenol-indophenol (DCIP) at 600 nm in a reaction mixture of 25 mM potassium phosphate (pH 7.5), 20 mM succinate, 0.5 mM DCIP, 10 μM rotenone, 2 μg/mL antimycin A, and 2 mM potassium cyanide. Complex III activity (Ubiquinol:cytochrome c oxidoreductase) was determined by measuring the reduction of cytochrome c at 550 nm in a reaction mixture of 25 mM potassium phosphate (pH 7.5), 35 μM reduced decylubiquinone, 15 μM cytochrome c, 10 μM rotenone, and 2 mM potassium cyanide. All activities were calculated as nmoles/min/mg protein and normalized to citrate synthase activity.

### 4.6. Tissue Preparation for Histological Studies

Seventy-two hours following SE, the animals were perfused with ice cold phosphate buffered saline (PBS, pH 7.4), followed by 2% paraformaldehyde in phosphate buffer (0.1 M PB). The brains were placed in 10% neutral buffered formalin for two days, followed by 30% sucrose, and cryopreservation. Serial coronal sections (30 μm) were obtained either through the anterior (from Bregma: −1.22 mm to −2.54 mm) or the posterior (from Bregma: −1.94 mm to −3.26 mm) hippocampus. One coronally-sectioned brain generated 4 series of 5 slides, each slide containing two 30 μm thick sections that are 120 μm apart. A given series was used for Fluoro-Jade B (FJ-B) or Nissl staining.

### 4.7. Fluoro-Jade B (FJ-B) Staining

Cryosections underwent serial rehydration steps in 100% and 70% EtOH, followed by immersion in 0.06% KMnO_4_ for 15 min. Subsequently, the sections were immersed in 0.001% FJ-B solution containing 4′, 6-diamidino-2-phenylindole (DAPI) for 30 min in the dark and air-dried overnight. Fluorescent images were obtained in serial coronal sections (10 s/animal) using a Nikon Elipse Ti-S microscope.

### 4.8. Stereological Estimates of the Hippocampal Neurons

Randomly selected series were stained with cresyl violet. An investigator who was blinded to the group assignment estimated neuronal number using the optical fractionator method with Stereo Investigator (MBF Bioscience, Williston, VT, USA). Systemic random sampling of hippocampal CA_1_ and CA_3_ regions was performed. A 25 μm × 25 μm or 50 μm × 50 μm counting frame was overlaid onto the high-power field (100× objective lens). Pyramidal neurons with discernible nuclei were counted if the nuclei were located within or in contact with the zone of inclusion. Total neuronal number (N) was estimated using the formula: N = ∑Q^−^ × (t/h) × (1/asf) × (1/ssf), where ∑Q^−^ is the number of neurons counted in the dissectors that fall within the counting frame, t is the section thickness (30 μm), h is the dissector height determined at individual sampling site, asf is the areal sampling fraction (area of the counting frame/area of the hippocampal subfield in a given coronal section), and ssf is the sectional sampling fraction (in this case = 4). The area of the hippocampal subfield was estimated using the Cavalieri method [37]. The adequacy of sampling was determined by estimating the coefficient of error (CE). Sampling was considered to be adequate when CE is ≤0.06 [37].

### 4.9. Statistical Analysis

PARP-1 activity was extrapolated from the standard curve and expressed as units of PARP/mg of protein. Western blot for pADPr-modified proteins was quantified using relative optical density (ROD) x area, summated, and normalized to actin on the same lane [29]. Two measurements were obtained for each mitochondrial O_2_ consumption experiment: (1) rate of O_2_ consumption in the presence of all substrates (state 3 respiration); (2) the rate of O_2_ consumption when ADP was depleted (state 4 respiration). The integrity of mitochondrial O_2_ consumption was evaluated by the respiratory control ratio (RCR), defined as a ratio of state 3 and state 4. Continuous data were analyzed using Analysis of Variance (ANOVA) with *post hoc* Tukey or Student *t*-test and expressed as mean ± SEM.
